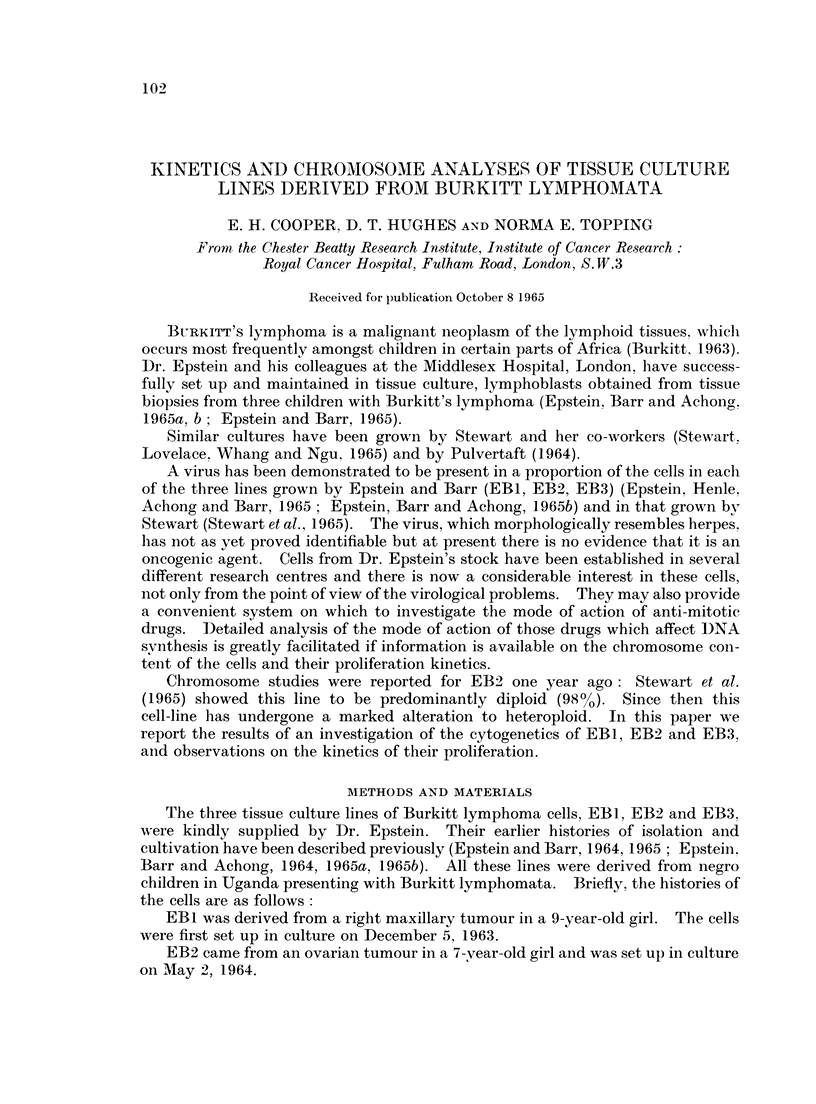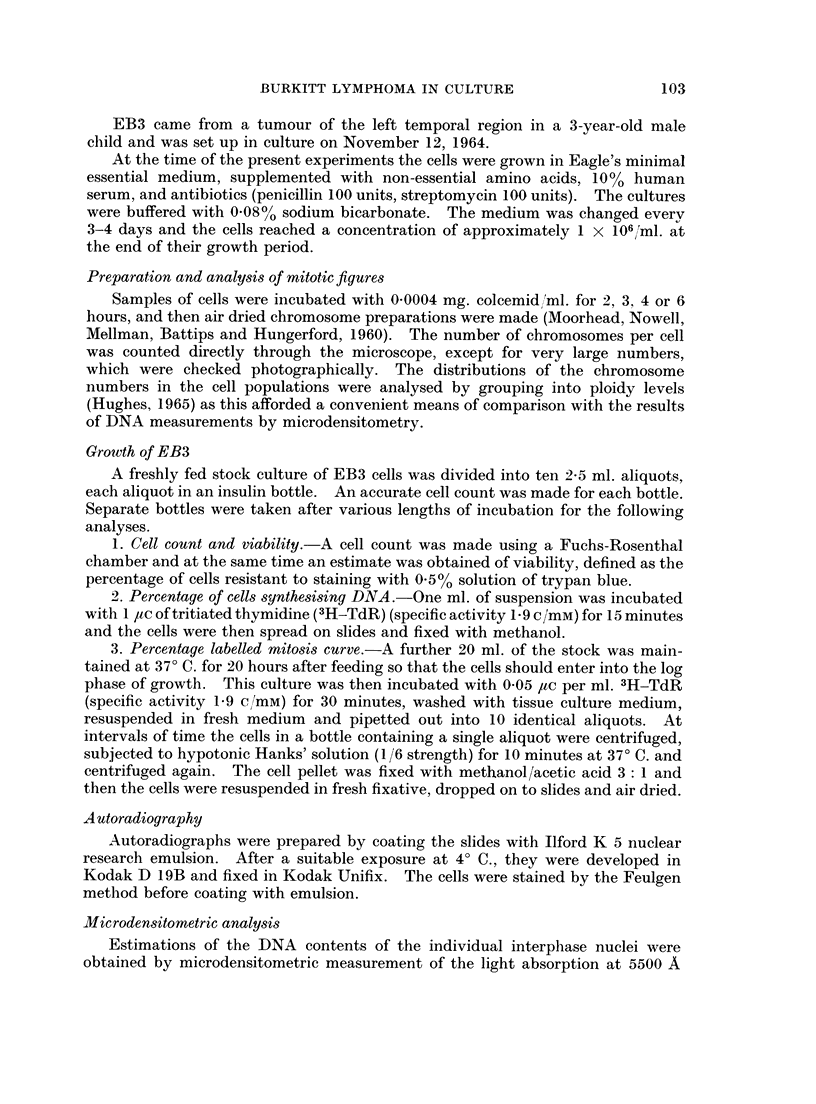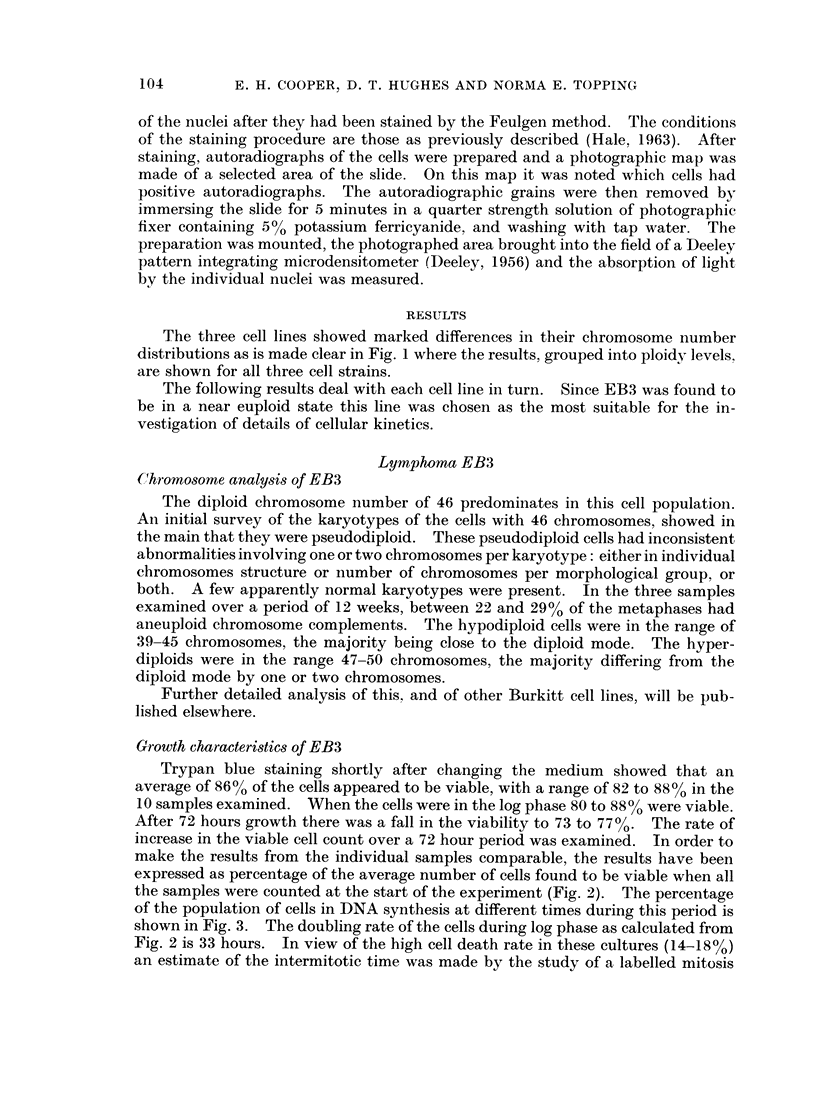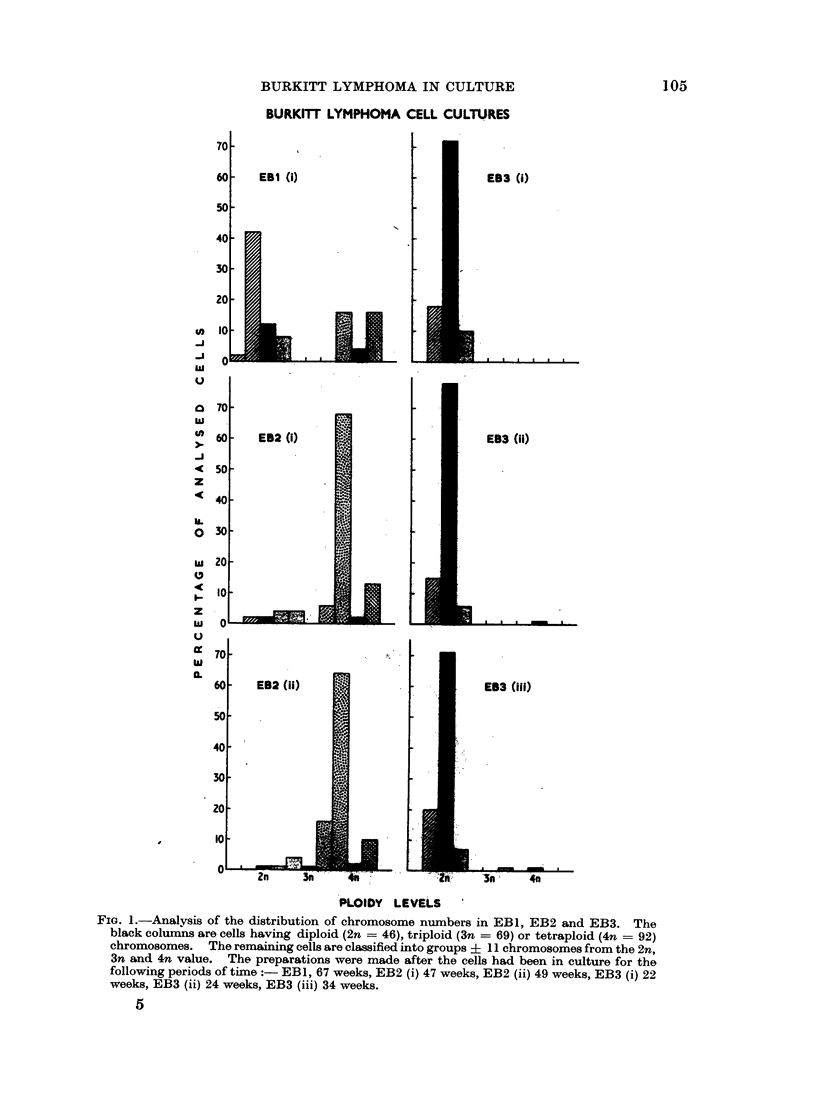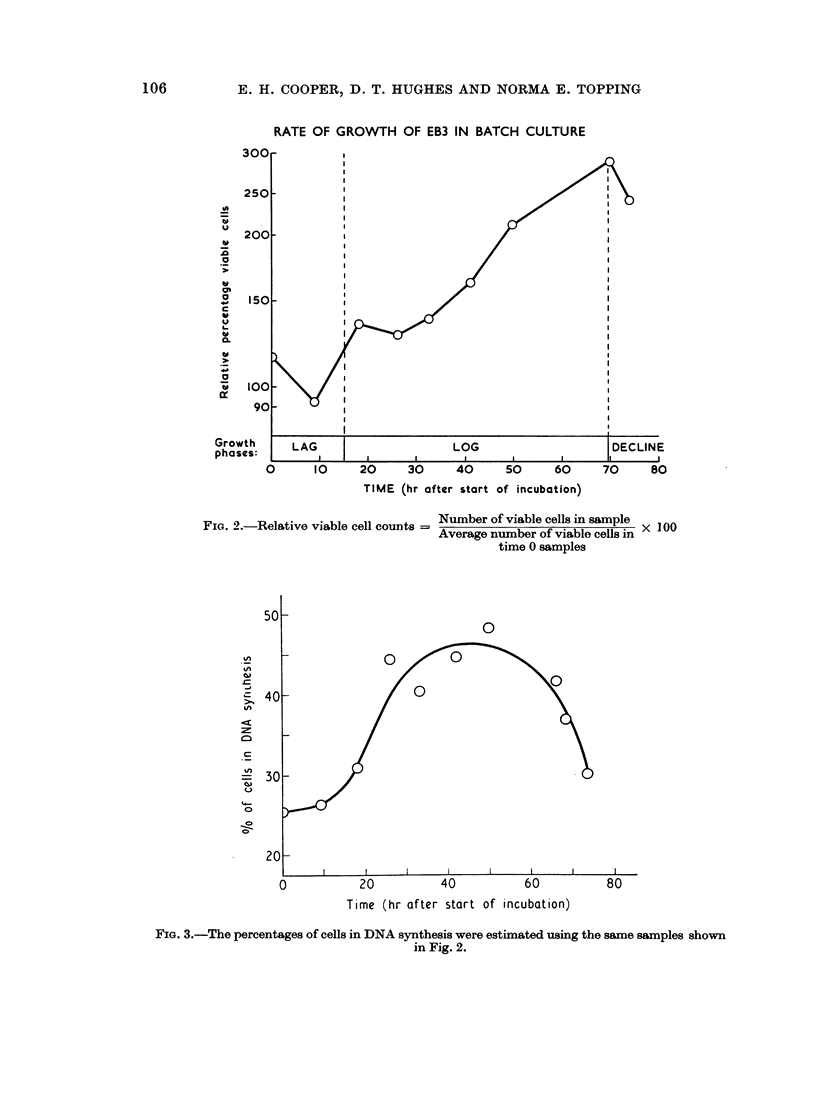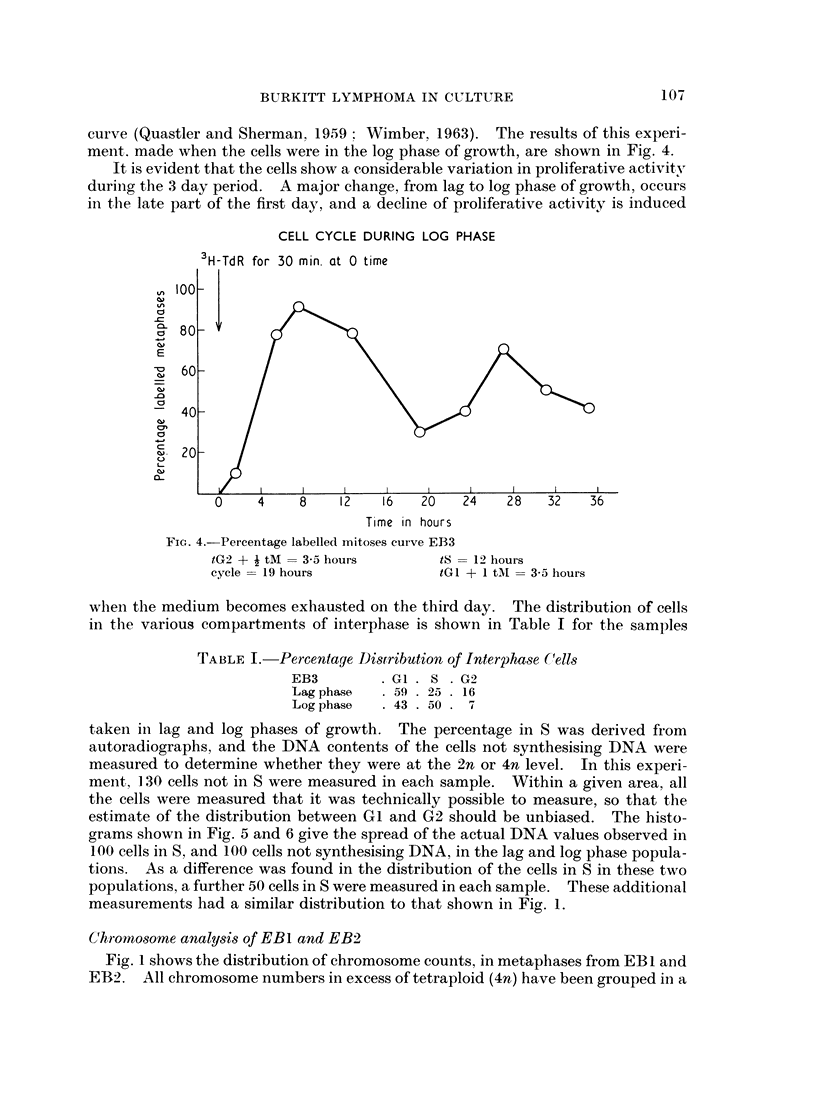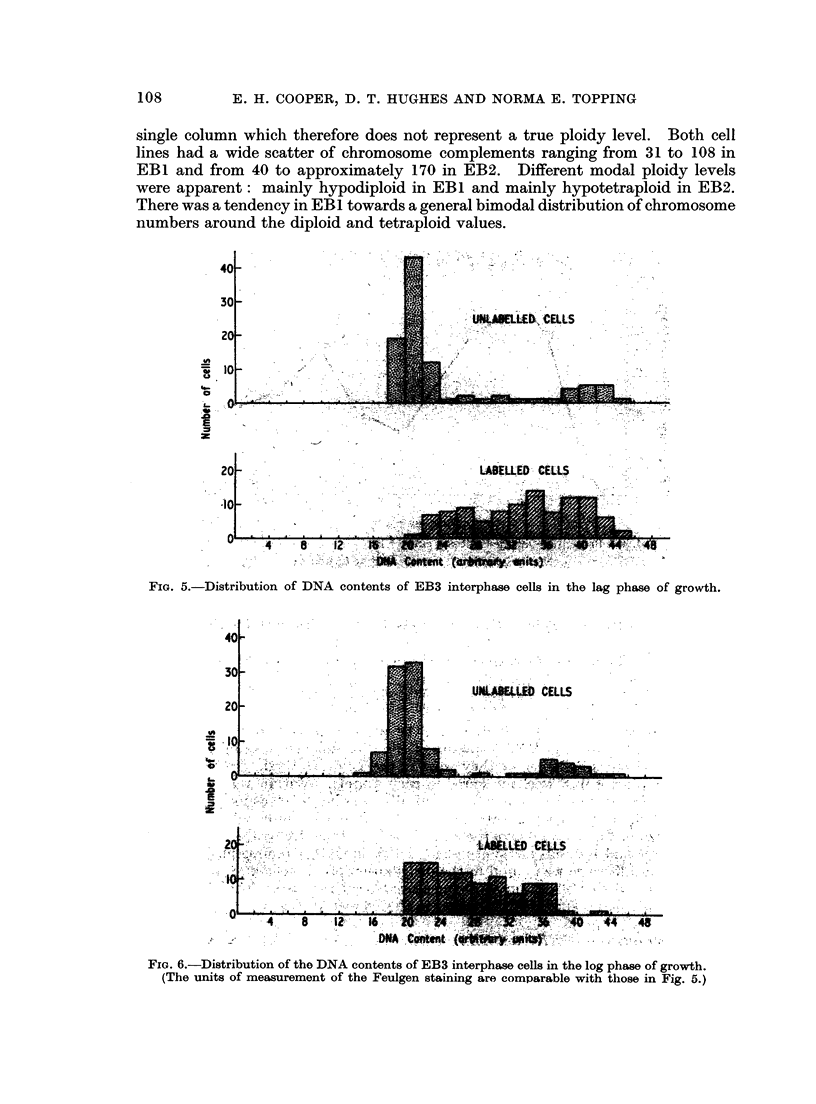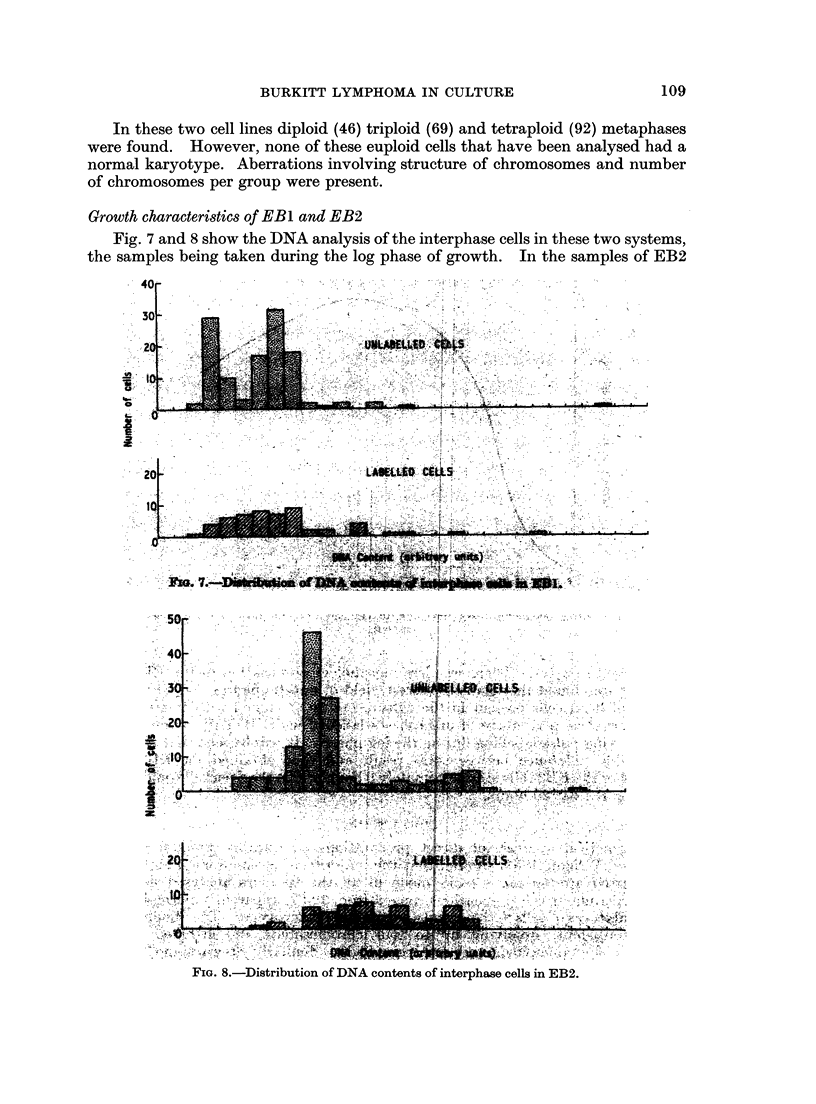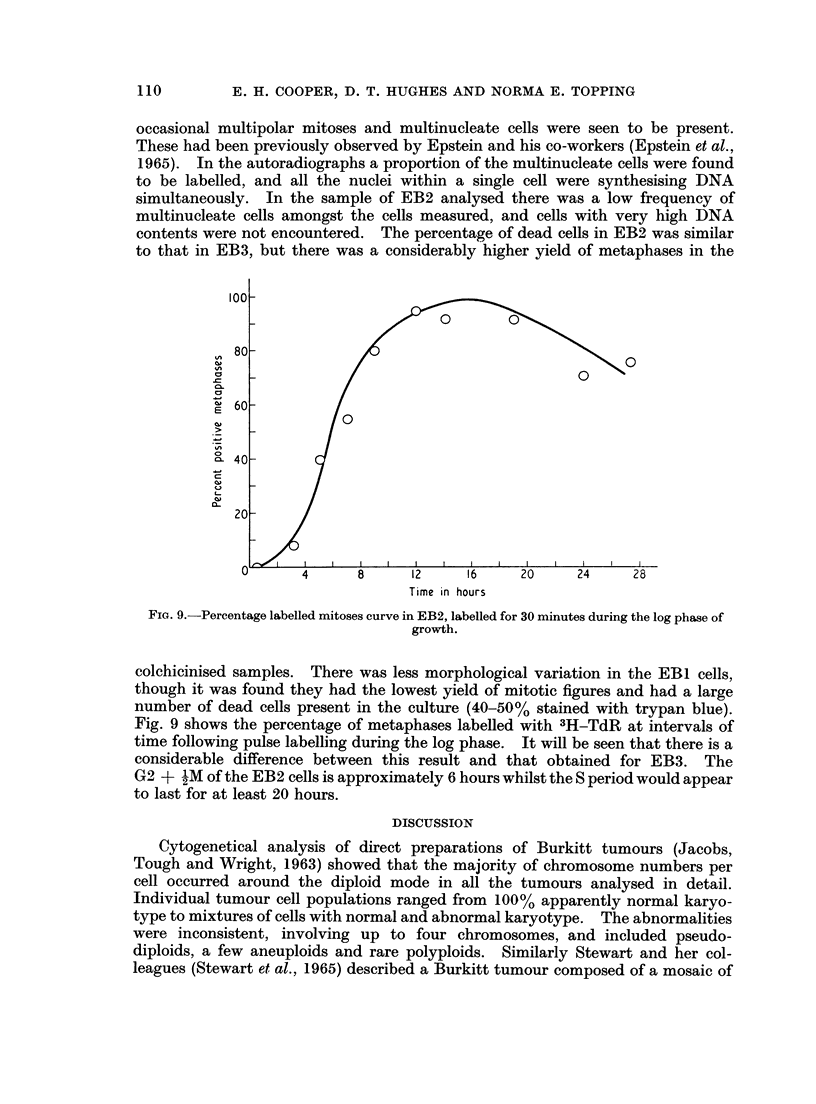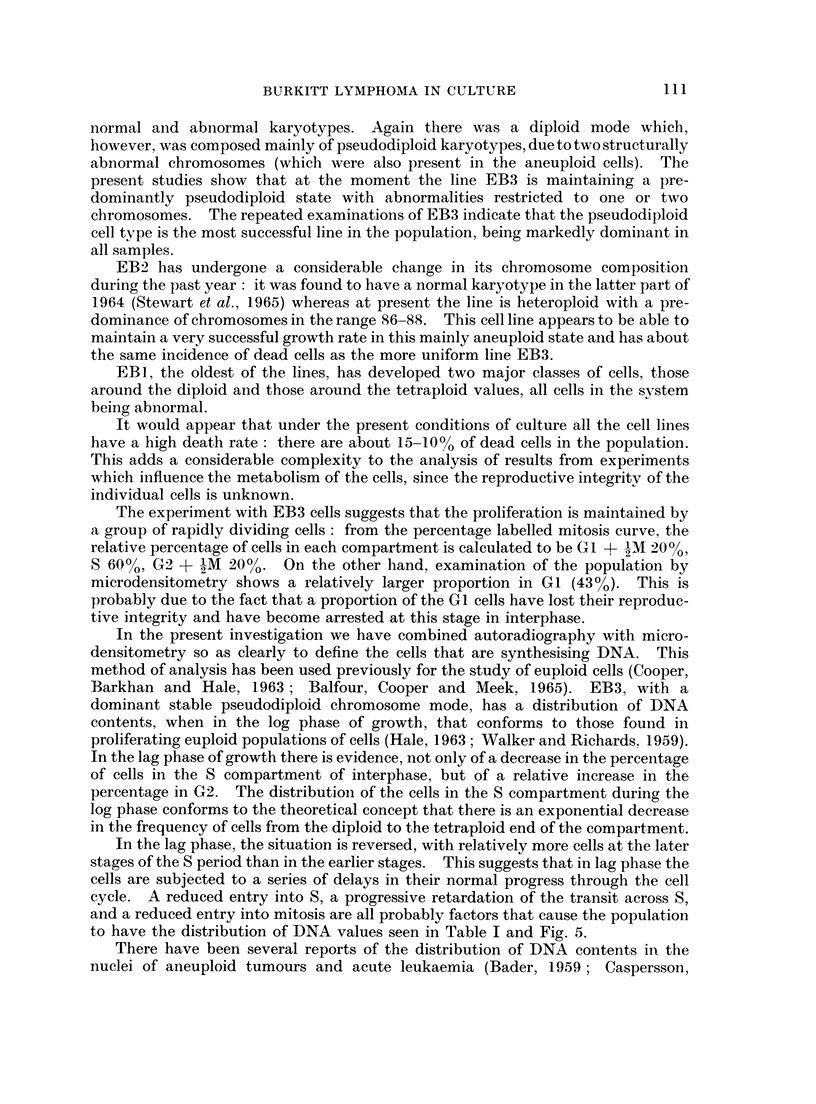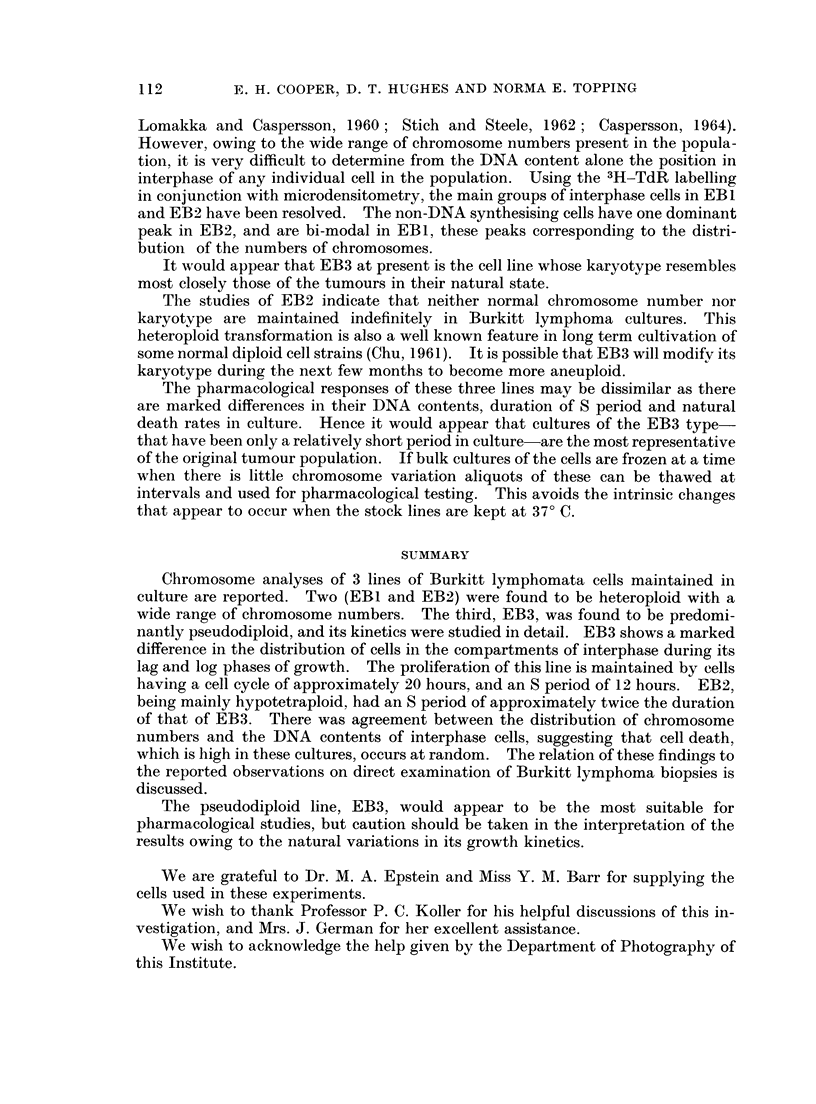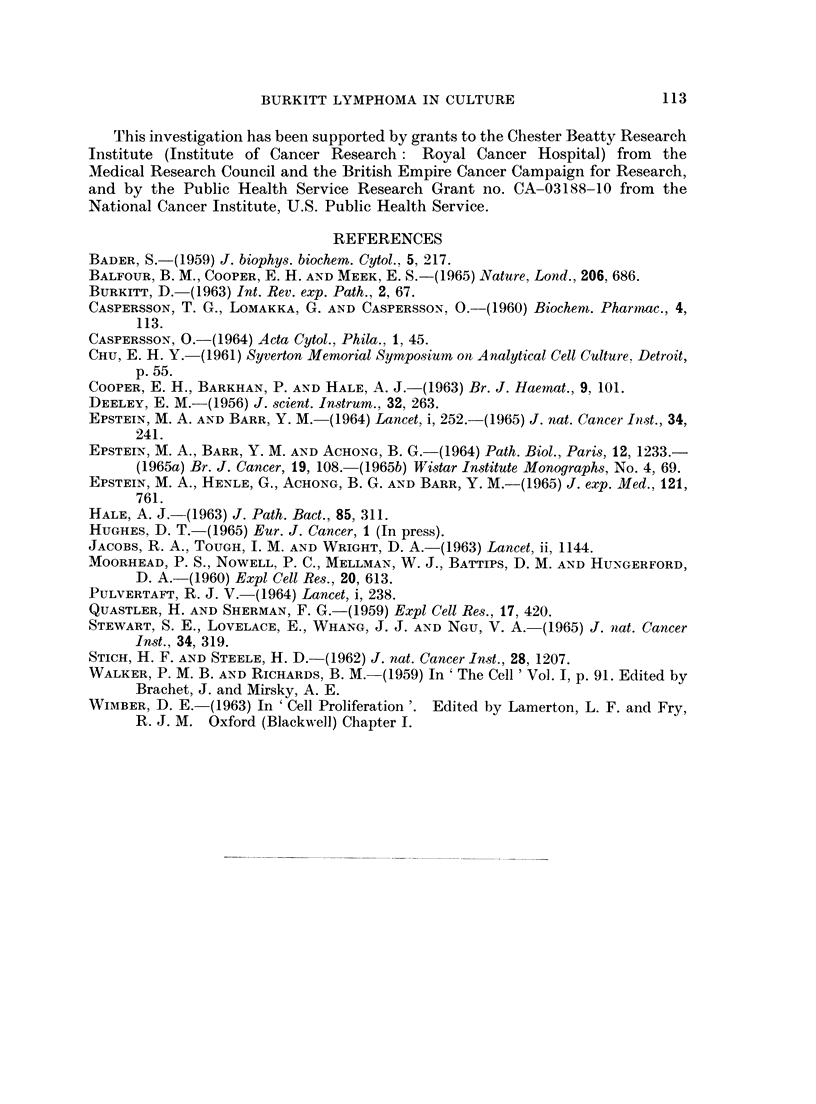# Kinetics and chromosome analyses of tissue culture lines derived from Burkitt lymphomata.

**DOI:** 10.1038/bjc.1966.11

**Published:** 1966-03

**Authors:** E. H. Cooper, D. T. Hughes, N. E. Topping


### KINETICS AND CHROMOSOME ANALYSES OF TISSUE CULTURE LINES DERIVED FROM BURKITT LYMPHOMATA

E. H. COOPER, D. T. HUGHES AND NORMA E. TOPPING

*From the Chester Beatty Research Institute, Institute of Cancer Research : Royal Cancer Hospital, Fulham Road, London, S.W.3*

Received for publication October 8 1965

BURKITT's lymphoma is a malignant neoplasm of the lymphoid tissues, which occurs most frequently amongst children in certain parts of Africa (Burkitt, 1963). Dr. Epstein and his colleagues at the Middlesex Hospital, London, have successfully set up and maintained in tissue culture, lymphoblasts obtained from tissue biopsies from three children with Burkitt's lymphoma (Epstein, Barr and Achong, 1965*a*, *b* ; Epstein and Barr, 1965).

Similar cultures have been grown by Stewart and her co-workers (Stewart, Lovelace, Whang and Ngu, 1965) and by Pulvertaft (1964).

A virus has been demonstrated to be present in a proportion of the cells in each of the three lines grown by Epstein and Barr (EB1, EB2, EB3) (Epstein, Henle, Achong and Barr, 1965 ; Epstein, Barr and Achong, 1965*b*) and in that grown by Stewart (Stewart *et al.*, 1965). The virus, which morphologically resembles herpes, has not as yet proved identifiable but at present there is no evidence that it is an oncogenic agent. Cells from Dr. Epstein's stock have been established in several different research centres and there is now a considerable interest in these cells, not only from the point of view of the virological problems. They may also provide a convenient system on which to investigate the mode of action of anti-mitotic drugs. Detailed analysis of the mode of action of those drugs which affect DNA synthesis is greatly facilitated if information is available on the chromosome content of the cells and their proliferation kinetics.

Chromosome studies were reported for EB2 one year ago : Stewart *et al.* (1965) showed this line to be predominantly diploid (98%). Since then this cell-line has undergone a marked alteration to heteroploid. In this paper we report the results of an investigation of the cytogenetics of EB1, EB2 and EB3, and observations on the kinetics of their proliferation.

### METHODS AND MATERIALS

The three tissue culture lines of Burkitt lymphoma cells, EB1, EB2 and EB3, were kindly supplied by Dr. Epstein. Their earlier histories of isolation and cultivation have been described previously (Epstein and Barr, 1964, 1965 ; Epstein, Barr and Achong, 1964, 1965*a*, 1965*b*). All these lines were derived from negro children in Uganda presenting with Burkitt lymphomata. Briefly, the histories of the cells are as follows :

EB1 was derived from a right maxillary tumour in a 9-year-old girl. The cells were first set up in culture on December 5, 1963.

EB2 came from an ovarian tumour in a 7-year-old girl and was set up in culture on May 2, 1964.

EB3 came from a tumour of the left temporal region in a 3-year-old male child and was set up in culture on November 12, 1964.

At the time of the present experiments the cells were grown in Eagle's minimal essential medium, supplemented with non-essential amino acids, 10% human serum, and antibiotics (penicillin 100 units, streptomycin 100 units). The cultures were buffered with 0·08% sodium bicarbonate. The medium was changed every 3–4 days and the cells reached a concentration of approximately $1 \times 10^6$/ml. at the end of their growth period.

*Preparation and analysis of mitotic figures*

Samples of cells were incubated with 0·0004 mg. colcemid/ml. for 2, 3, 4 or 6 hours, and then air dried chromosome preparations were made (Moorhead, Nowell, Mellman, Battips and Hungerford, 1960). The number of chromosomes per cell was counted directly through the microscope, except for very large numbers, which were checked photographically. The distributions of the chromosome numbers in the cell populations were analysed by grouping into ploidy levels (Hughes, 1965) as this afforded a convenient means of comparison with the results of DNA measurements by microdensitometry.

*Growth of EB3*

A freshly fed stock culture of EB3 cells was divided into ten 2·5 ml. aliquots, each aliquot in an insulin bottle. An accurate cell count was made for each bottle. Separate bottles were taken after various lengths of incubation for the following analyses.

1. *Cell count and viability.*—A cell count was made using a Fuchs-Rosenthal chamber and at the same time an estimate was obtained of viability, defined as the percentage of cells resistant to staining with 0·5% solution of trypan blue.

2. *Percentage of cells synthesising DNA.*—One ml. of suspension was incubated with 1 μc of tritiated thymidine ($^3$H–TdR) (specific activity 1·9 c/mM) for 15 minutes and the cells were then spread on slides and fixed with methanol.

3. *Percentage labelled mitosis curve.*—A further 20 ml. of the stock was maintained at 37° C. for 20 hours after feeding so that the cells should enter into the log phase of growth. This culture was then incubated with 0·05 μc per ml. $^3$H–TdR (specific activity 1·9 c/mM) for 30 minutes, washed with tissue culture medium, resuspended in fresh medium and pipetted out into 10 identical aliquots. At intervals of time the cells in a bottle containing a single aliquot were centrifuged, subjected to hypotonic Hanks' solution (1/6 strength) for 10 minutes at 37° C. and centrifuged again. The cell pellet was fixed with methanol/acetic acid 3 : 1 and then the cells were resuspended in fresh fixative, dropped on to slides and air dried.

*Autoradiography*

Autoradiographs were prepared by coating the slides with Ilford K 5 nuclear research emulsion. After a suitable exposure at 4° C., they were developed in Kodak D 19B and fixed in Kodak Unifix. The cells were stained by the Feulgen method before coating with emulsion.

*Microdensitometric analysis*

Estimations of the DNA contents of the individual interphase nuclei were obtained by microdensitometric measurement of the light absorption at 5500 Å

of the nuclei after they had been stained by the Feulgen method. The conditions of the staining procedure are those as previously described (Hale, 1963). After staining, autoradiographs of the cells were prepared and a photographic map was made of a selected area of the slide. On this map it was noted which cells had positive autoradiographs. The autoradiographic grains were then removed by immersing the slide for 5 minutes in a quarter strength solution of photographic fixer containing 5% potassium ferricyanide, and washing with tap water. The preparation was mounted, the photographed area brought into the field of a Deeley pattern integrating microdensitometer (Deeley, 1956) and the absorption of light by the individual nuclei was measured.

### RESULTS

The three cell lines showed marked differences in their chromosome number distributions as is made clear in Fig. 1 where the results, grouped into ploidy levels, are shown for all three cell strains.

The following results deal with each cell line in turn. Since EB3 was found to be in a near euploid state this line was chosen as the most suitable for the investigation of details of cellular kinetics.

### *Lymphoma EB3*

#### *Chromosome analysis of EB3*

The diploid chromosome number of 46 predominates in this cell population. An initial survey of the karyotypes of the cells with 46 chromosomes, showed in the main that they were pseudodiploid. These pseudodiploid cells had inconsistent abnormalities involving one or two chromosomes per karyotype: either in individual chromosomes structure or number of chromosomes per morphological group, or both. A few apparently normal karyotypes were present. In the three samples examined over a period of 12 weeks, between 22 and 29% of the metaphases had aneuploid chromosome complements. The hypodiploid cells were in the range of 39–45 chromosomes, the majority being close to the diploid mode. The hyperdiploids were in the range 47–50 chromosomes, the majority differing from the diploid mode by one or two chromosomes.

Further detailed analysis of this, and of other Burkitt cell lines, will be published elsewhere.

#### *Growth characteristics of EB3*

Trypan blue staining shortly after changing the medium showed that an average of 86% of the cells appeared to be viable, with a range of 82 to 88% in the 10 samples examined. When the cells were in the log phase 80 to 88% were viable. After 72 hours growth there was a fall in the viability to 73 to 77%. The rate of increase in the viable cell count over a 72 hour period was examined. In order to make the results from the individual samples comparable, the results have been expressed as percentage of the average number of cells found to be viable when all the samples were counted at the start of the experiment (Fig. 2). The percentage of the population of cells in DNA synthesis at different times during this period is shown in Fig. 3. The doubling rate of the cells during log phase as calculated from Fig. 2 is 33 hours. In view of the high cell death rate in these cultures (14–18%) an estimate of the intermitotic time was made by the study of a labelled mitosis

FIG. 1.—Analysis of the distribution of chromosome numbers in EB1, EB2 and EB3. The black columns are cells having diploid ($2n = 46$), triploid ($3n = 69$) or tetraploid ($4n = 92$) chromosomes. The remaining cells are classified into groups $\pm$ 11 chromosomes from the $2n$, $3n$ and $4n$ value. The preparations were made after the cells had been in culture for the following periods of time :— EB1, 67 weeks, EB2 (i) 47 weeks, EB2 (ii) 49 weeks, EB3 (i) 22 weeks, EB3 (ii) 24 weeks, EB3 (iii) 34 weeks.

FIG. 2.—Relative viable cell counts $= \frac{\text{Number of viable cells in sample}}{\text{Average number of viable cells in time 0 samples}} \times 100$

FIG. 3.—The percentages of cells in DNA synthesis were estimated using the same samples shown in Fig. 2.

curve (Quastler and Sherman, 1959; Wimber, 1963). The results of this experiment, made when the cells were in the log phase of growth, are shown in Fig. 4.

It is evident that the cells show a considerable variation in proliferative activity during the 3 day period. A major change, from lag to log phase of growth, occurs in the late part of the first day, and a decline of proliferative activity is induced

FIG. 4.—Percentage labelled mitoses curve EB3

*t*G2 + ½ tM = 3·5 hours    *t*S = 12 hours cycle = 19 hours    *t*G1 + 1 tM = 3·5 hours when the medium becomes exhausted on the third day. The distribution of cells in the various compartments of interphase is shown in Table I for the samples

TABLE I.—*Percentage Distribution of Interphase Cells*

| EB3 | G1 | S | G2 |
|---|---|---|---|
| Lag phase | 59 | 25 | 16 |
| Log phase | 43 | 50 | 7 |

taken in lag and log phases of growth. The percentage in S was derived from autoradiographs, and the DNA contents of the cells not synthesising DNA were measured to determine whether they were at the $2n$ or $4n$ level. In this experiment, 130 cells not in S were measured in each sample. Within a given area, all the cells were measured that it was technically possible to measure, so that the estimate of the distribution between G1 and G2 should be unbiased. The histograms shown in Fig. 5 and 6 give the spread of the actual DNA values observed in 100 cells in S, and 100 cells not synthesising DNA, in the lag and log phase populations. As a difference was found in the distribution of the cells in S in these two populations, a further 50 cells in S were measured in each sample. These additional measurements had a similar distribution to that shown in Fig. 1.

*Chromosome analysis of EB1 and EB2*

Fig. 1 shows the distribution of chromosome counts, in metaphases from EB1 and EB2. All chromosome numbers in excess of tetraploid ($4n$) have been grouped in a single column which therefore does not represent a true ploidy level. Both cell lines had a wide scatter of chromosome complements ranging from 31 to 108 in EB1 and from 40 to approximately 170 in EB2. Different modal ploidy levels were apparent: mainly hypodiploid in EB1 and mainly hypotetraploid in EB2. There was a tendency in EB1 towards a general bimodal distribution of chromosome numbers around the diploid and tetraploid values.

FIG. 5.—Distribution of DNA contents of EB3 interphase cells in the lag phase of growth.

FIG. 6.—Distribution of the DNA contents of EB3 interphase cells in the log phase of growth. (The units of measurement of the Feulgen staining are comparable with those in Fig. 5.)

In these two cell lines diploid (46) triploid (69) and tetraploid (92) metaphases were found. However, none of these euploid cells that have been analysed had a normal karyotype. Aberrations involving structure of chromosomes and number of chromosomes per group were present.

*Growth characteristics of EB1 and EB2*

Fig. 7 and 8 show the DNA analysis of the interphase cells in these two systems, the samples being taken during the log phase of growth. In the samples of EB2

FIG. 7.—Distribution of DNA contents of interphase cells in EB1.

FIG. 8.—Distribution of DNA contents of interphase cells in EB2.

occasional multipolar mitoses and multinucleate cells were seen to be present. These had been previously observed by Epstein and his co-workers (Epstein *et al.*, 1965). In the autoradiographs a proportion of the multinucleate cells were found to be labelled, and all the nuclei within a single cell were synthesising DNA simultaneously. In the sample of EB2 analysed there was a low frequency of multinucleate cells amongst the cells measured, and cells with very high DNA contents were not encountered. The percentage of dead cells in EB2 was similar to that in EB3, but there was a considerably higher yield of metaphases in the

FIG. 9.—Percentage labelled mitoses curve in EB2, labelled for 30 minutes during the log phase of growth.

colchicinised samples. There was less morphological variation in the EB1 cells, though it was found they had the lowest yield of mitotic figures and had a large number of dead cells present in the culture (40–50% stained with trypan blue). Fig. 9 shows the percentage of metaphases labelled with $^3$H–TdR at intervals of time following pulse labelling during the log phase. It will be seen that there is a considerable difference between this result and that obtained for EB3. The G2 + $\frac{1}{2}$M of the EB2 cells is approximately 6 hours whilst the S period would appear to last for at least 20 hours.

### DISCUSSION

Cytogenetical analysis of direct preparations of Burkitt tumours (Jacobs, Tough and Wright, 1963) showed that the majority of chromosome numbers per cell occurred around the diploid mode in all the tumours analysed in detail. Individual tumour cell populations ranged from 100% apparently normal karyotype to mixtures of cells with normal and abnormal karyotype. The abnormalities were inconsistent, involving up to four chromosomes, and included pseudodiploids, a few aneuploids and rare polyploids. Similarly Stewart and her colleagues (Stewart *et al.*, 1965) described a Burkitt tumour composed of a mosaic of normal and abnormal karyotypes. Again there was a diploid mode which, however, was composed mainly of pseudodiploid karyotypes, due to two structurally abnormal chromosomes (which were also present in the aneuploid cells). The present studies show that at the moment the line EB3 is maintaining a predominantly pseudodiploid state with abnormalities restricted to one or two chromosomes. The repeated examinations of EB3 indicate that the pseudodiploid cell type is the most successful line in the population, being markedly dominant in all samples.

EB2 has undergone a considerable change in its chromosome composition during the past year : it was found to have a normal karyotype in the latter part of 1964 (Stewart *et al.*, 1965) whereas at present the line is heteroploid with a predominance of chromosomes in the range 86–88. This cell line appears to be able to maintain a very successful growth rate in this mainly aneuploid state and has about the same incidence of dead cells as the more uniform line EB3.

EB1, the oldest of the lines, has developed two major classes of cells, those around the diploid and those around the tetraploid values, all cells in the system being abnormal.

It would appear that under the present conditions of culture all the cell lines have a high death rate : there are about 15–10% of dead cells in the population. This adds a considerable complexity to the analysis of results from experiments which influence the metabolism of the cells, since the reproductive integrity of the individual cells is unknown.

The experiment with EB3 cells suggests that the proliferation is maintained by a group of rapidly dividing cells : from the percentage labelled mitosis curve, the relative percentage of cells in each compartment is calculated to be G1 + $\frac{1}{2}$M 20%, S 60%, G2 + $\frac{1}{2}$M 20%. On the other hand, examination of the population by microdensitometry shows a relatively larger proportion in G1 (43%). This is probably due to the fact that a proportion of the G1 cells have lost their reproductive integrity and have become arrested at this stage in interphase.

In the present investigation we have combined autoradiography with microdensitometry so as clearly to define the cells that are synthesising DNA. This method of analysis has been used previously for the study of euploid cells (Cooper, Barkhan and Hale, 1963 ; Balfour, Cooper and Meek, 1965). EB3, with a dominant stable pseudodiploid chromosome mode, has a distribution of DNA contents, when in the log phase of growth, that conforms to those found in proliferating euploid populations of cells (Hale, 1963 ; Walker and Richards, 1959). In the lag phase of growth there is evidence, not only of a decrease in the percentage of cells in the S compartment of interphase, but of a relative increase in the percentage in G2. The distribution of the cells in the S compartment during the log phase conforms to the theoretical concept that there is an exponential decrease in the frequency of cells from the diploid to the tetraploid end of the compartment.

In the lag phase, the situation is reversed, with relatively more cells at the later stages of the S period than in the earlier stages. This suggests that in lag phase the cells are subjected to a series of delays in their normal progress through the cell cycle. A reduced entry into S, a progressive retardation of the transit across S, and a reduced entry into mitosis are all probably factors that cause the population to have the distribution of DNA values seen in Table I and Fig. 5.

There have been several reports of the distribution of DNA contents in the nuclei of aneuploid tumours and acute leukaemia (Bader, 1959 ; Caspersson,

Lomakka and Caspersson, 1960; Stich and Steele, 1962; Caspersson, 1964). However, owing to the wide range of chromosome numbers present in the population, it is very difficult to determine from the DNA content alone the position in interphase of any individual cell in the population. Using the $^3$H–TdR labelling in conjunction with microdensitometry, the main groups of interphase cells in EB1 and EB2 have been resolved. The non-DNA synthesising cells have one dominant peak in EB2, and are bi-modal in EB1, these peaks corresponding to the distribution of the numbers of chromosomes.

It would appear that EB3 at present is the cell line whose karyotype resembles most closely those of the tumours in their natural state.

The studies of EB2 indicate that neither normal chromosome number nor karyotype are maintained indefinitely in Burkitt lymphoma cultures. This heteroploid transformation is also a well known feature in long term cultivation of some normal diploid cell strains (Chu, 1961). It is possible that EB3 will modify its karyotype during the next few months to become more aneuploid.

The pharmacological responses of these three lines may be dissimilar as there are marked differences in their DNA contents, duration of S period and natural death rates in culture. Hence it would appear that cultures of the EB3 type—that have been only a relatively short period in culture—are the most representative of the original tumour population. If bulk cultures of the cells are frozen at a time when there is little chromosome variation aliquots of these can be thawed at intervals and used for pharmacological testing. This avoids the intrinsic changes that appear to occur when the stock lines are kept at 37° C.

### SUMMARY

Chromosome analyses of 3 lines of Burkitt lymphomata cells maintained in culture are reported. Two (EB1 and EB2) were found to be heteroploid with a wide range of chromosome numbers. The third, EB3, was found to be predominantly pseudodiploid, and its kinetics were studied in detail. EB3 shows a marked difference in the distribution of cells in the compartments of interphase during its lag and log phases of growth. The proliferation of this line is maintained by cells having a cell cycle of approximately 20 hours, and an S period of 12 hours. EB2, being mainly hypotetraploid, had an S period of approximately twice the duration of that of EB3. There was agreement between the distribution of chromosome numbers and the DNA contents of interphase cells, suggesting that cell death, which is high in these cultures, occurs at random. The relation of these findings to the reported observations on direct examination of Burkitt lymphoma biopsies is discussed.

The pseudodiploid line, EB3, would appear to be the most suitable for pharmacological studies, but caution should be taken in the interpretation of the results owing to the natural variations in its growth kinetics.

We are grateful to Dr. M. A. Epstein and Miss Y. M. Barr for supplying the cells used in these experiments.

We wish to thank Professor P. C. Koller for his helpful discussions of this investigation, and Mrs. J. German for her excellent assistance.

We wish to acknowledge the help given by the Department of Photography of this Institute.

This investigation has been supported by grants to the Chester Beatty Research Institute (Institute of Cancer Research: Royal Cancer Hospital) from the Medical Research Council and the British Empire Cancer Campaign for Research, and by the Public Health Service Research Grant no. CA-03188-10 from the National Cancer Institute, U.S. Public Health Service.

### REFERENCES

BADER, S.—(1959) *J. biophys. biochem. Cytol.*, **5**, 217.
BALFOUR, B. M., COOPER, E. H. AND MEEK, E. S.—(1965) *Nature, Lond.*, **206**, 686.
BURKITT, D.—(1963) *Int. Rev. exp. Path.*, **2**, 67.
CASPERSSON, T. G., LOMAKKA, G. AND CASPERSSON, O.—(1960) *Biochem. Pharmac.*, **4**, 113.
CASPERSSON, O.—(1964) *Acta Cytol., Phila.*, **1**, 45.
CHU, E. H. Y.—(1961) *Syverton Memorial Symposium on Analytical Cell Culture, Detroit*, p. 55.
COOPER, E. H., BARKHAN, P. AND HALE, A. J.—(1963) *Br. J. Haemat.*, **9**, 101.
DEELEY, E. M.—(1956) *J. scient. Instrum.*, **32**, 263.
EPSTEIN, M. A. AND BARR, Y. M.—(1964) *Lancet*, i, 252.—(1965) *J. nat. Cancer Inst.*, **34**, 241.
EPSTEIN, M. A., BARR, Y. M. AND ACHONG, B. G.—(1964) *Path. Biol., Paris*, **12**, 1233.—(1965*a*) *Br. J. Cancer*, **19**, 108.—(1965*b*) *Wistar Institute Monographs*, No. 4, 69.
EPSTEIN, M. A., HENLE, G., ACHONG, B. G. AND BARR, Y. M.—(1965) *J. exp. Med.*, **121**, 761.
HALE, A. J.—(1963) *J. Path. Bact.*, **85**, 311.
HUGHES, D. T.—(1965) *Eur. J. Cancer*, **1** (In press).
JACOBS, R. A., TOUGH, I. M. AND WRIGHT, D. A.—(1963) *Lancet*, ii, 1144.
MOORHEAD, P. S., NOWELL, P. C., MELLMAN, W. J., BATTIPS, D. M. AND HUNGERFORD, D. A.—(1960) *Expl Cell Res.*, **20**, 613.
PULVERTAFT, R. J. V.—(1964) *Lancet*, i, 238.
QUASTLER, H. AND SHERMAN, F. G.—(1959) *Expl Cell Res.*, **17**, 420.
STEWART, S. E., LOVELACE, E., WHANG, J. J. AND NGU, V. A.—(1965) *J. nat. Cancer Inst.*, **34**, 319.
STICH, H. F. AND STEELE, H. D.—(1962) *J. nat. Cancer Inst.*, **28**, 1207.
WALKER, P. M. B. AND RICHARDS, B. M.—(1959) In ' The Cell ' Vol. I, p. 91. Edited by Brachet, J. and Mirsky, A. E.
WIMBER, D. E.—(1963) In ' Cell Proliferation '. Edited by Lamerton, L. F. and Fry, R. J. M. Oxford (Blackwell) Chapter I.